# A design framework and exemplar metrics for FAIRness

**DOI:** 10.1038/sdata.2018.118

**Published:** 2018-06-26

**Authors:** Mark D. Wilkinson, Susanna-Assunta Sansone, Erik Schultes, Peter Doorn, Luiz Olavo Bonino da Silva Santos, Michel Dumontier

**Affiliations:** 1 Centro de Biotecnología y Genómica de Plantas UPM – INIA, Madrid, Spain; 2 Oxford e-Research Centre, Department of Engineering Science, University of Oxford, Oxford, UK; 3 Dutch Techcentre for Life Sciences, Utrecht, The Netherlands; 4 Data Archiving and Networked Services, Den Haag, The Netherlands; 5 GO FAIR International Support and Coordination Office, Leiden, The Netherlands; 6 Leiden University Medical Centre, Leiden, The Netherlands; 7 Institute of Data Science, Maastricht University, Maastricht, The Netherlands

**Keywords:** Publication characteristics, Data publication and archiving

The FAIR Principles^1^ (https://doi.org/10.25504/FAIRsharing.WWI10U) provide guidelines for the publication of digital resources such as datasets, code, workflows, and research objects, in a manner that makes them Findable, Accessible, Interoperable, and Reusable (FAIR). The Principles have rapidly been adopted by publishers, funders, and pan-disciplinary infrastructure programmes and societies. The Principles are aspirational, in that they do not strictly define how to achieve a state of "FAIRness", but rather they describe a continuum of features, attributes, and behaviors that will move a digital resource closer to that goal. This ambiguity has led to a wide range of interpretations of FAIRness, with some resources even claiming to already "be FAIR"! The increasing number of such statements, the emergence of subjective and self-assessments of FAIRness^2,3^, and the need of data and service providers, journals, funding agencies, and regulatory bodies to qualitatively or quantitatively evaluate such claims, led us to self-assemble and establish a FAIR Metrics group (http://fairmetrics.org) to pursue the goal of defining ways to measure FAIRness.

As co-authors of the FAIR Principles and its associated manuscript, founding this small focus group was a natural and timely step for us, and we foresee group membership expanding and broadening according to the needs and enthusiasm of the various stakeholder communities. Nevertheless, in this first phase of group activities we did not work in isolation, but we gathered use cases and requirements from the communities, organizations and projects we are core members of, and where discussions on how to measure FAIRness have also started. Our community network and formal participation encompasses generic and discipline-specific initiatives, including: the Global and Open FAIR (http://go-fair.org), the European Open Science Cloud (EOSC; https://eoscpilot.eu), working groups of the Research Data Alliance (RDA; https://www.rd-alliance.org) and Force11 (https://www.force11.org), the Data Seal of Approval^4^, Nodes of the European ELIXIR infrastructure (https://www.elixir-europe.org), projects under the USA National Institutes of Health (NIH)’s Big Data to Knowledge Initiative (BD2K) and its new Data Commons Pilots (https://commonfund.nih.gov/bd2k/commons). In addition, via the FAIRsharing network and advisory board (https://fairsharing.org), we are also connected to open standards-developing communities and data policy leaders, and also editors and publishers, especially those very active around data matters, such as: Springer Nature’s *Scientific Data*, *Nature Genetics* and *BioMedCentral*, *PloS Biology, The BMJ*, Oxford University Press’s *GigaScience*, *F1000Research*, *Wellcome Open Research*, Elsevier, EMBO Press and Ubiquity Press.

The converging viewpoints on FAIR metrics and FAIRness, arising from our information-gathering discussions with these various communities and stakeholders groups, can be summarized as it follows:

- Metrics should address the multi-dimensionality of the FAIR principles, and encompass all types of digital objects.
- Universal metrics may be complemented by additional resource-specific metrics that reflect the expectations of particular communities.
- The metrics themselves, and any results stemming from their application, must be FAIR.
- Open standards around the metrics should foster a vibrant ecosystem of FAIRness assessment tools.
- Various approaches to FAIR assessment should be enabled (e.g. self-assessment, task forces, crowd-sourcing, automated), however, the ability to scale FAIRness assessments to billions if not trillions of diverse digital objects is critical.
- FAIRness assessments should be kept up to date, and all assessments should be versioned, have a time stamp, and be publicly accessible.
- FAIRness assessments presented as a simple visualization, will be a powerful modality to inform users and guide the work of producers of digital resources.
- The assessment process, and the resulting FAIRness assessment, should be designed and disseminated in a manner that positively incentivizes the providers of digital resources; i.e., they should view the process as being fair and unbiased, and moreover, should benefit from these assessments and use them as an opportunity to identify areas of improvement.
- Governance over the metrics, and the mechanisms for assessing them, will be required to enable their careful evolution and address valid disagreements.

Here we report on the framework we have developed, which encompasses the first iteration of a core set of FAIRness indicators that can be objectively measured by a semi-automated process, and a template that can be followed within individual scholarly domains to derive community-specific metrics evaluating FAIR aspects important to them.

From the outset, the group decided that it would focus on FAIRness for machines – i.e., the degree to which a digital resource is findable, accessible, interoperable, and reusable without human intervention. This was because FAIRness for people would be difficult to measure objectively, as it would often depend on the experience and prior-knowledge of the individual attempting to find and access the data. We further agreed on the qualities that a FAIR metric should exhibit. A good metric should be:

- Clear: anyone can understand the purpose of the metric
- Realistic: it should not be unduly complicated for a resource to comply with the metric
- Discriminating: the metric should measure something important for FAIRness; distinguish the degree to which that resource meets that objective; and be able to provide instruction as to what would maximize that value
- Measurable: the assessment can be made in an objective, quantitative, machine-interpretable, scalable and reproducible manner, ensuring transparency of what is being measured, and how.
- Universal: The metric should be applicable to all digital resources.

The goal of this working group was to derive at least one metric for each of the FAIR sub-principles that would be universally applicable to all digital resources in all scholarly domains. We recognized, however, that what is considered FAIR in one community may be quite different from the FAIRness requirements or expectations in another community – different community norms, standards, and practices make this a certainty. As such, our approach took into account that the metrics we derived would eventually be supplemented by individual community members through the creation of domain-specific or community-specific metrics. With this in mind, we developed (and utilized) a template for the creation of metrics (Table 1), that we suggest should be followed by communities who engage in this process.

The outcome of this process was 14 exemplar universal metrics covering each of the FAIR sub-principles (the short names of the metrics are in brackets in the following description). The metrics request a variety of evidence from the community, some of which may require specific new actions. For instance, digital resource providers must provide a publicly accessible document(s) that provides machine-readable metadata (FM-F2, FM-F3) and details their plans with respect to identifier management (FM-F1B), metadata longevity (FM-A2), and any additional authorization procedures (FM-A1.2). They must ensure the public registration of their identifier schemes (FM-F1A), (secure) access protocols (FM-A1.1), knowledge representation languages (FM-I1), licenses (FM-R1.1), provenance specifications (FM-R1.2). Evidence of ability to find the digital resource in search results (FM-F4), linking to other resources (FM-I3), FAIRness of linked resources (FM-I2), and meeting community standards (FM-R1.3) must also be provided. The current metrics are available for public discussion at the FAIR Metrics GitHub, with suggestions and comments being made through the GitHub comment submission system (https://github.com/FAIRMetrics). They are free to use for any purpose under the CC0 license. Versioned releases will be made to Zenodo as the metrics evolve, with the first release already available for download^5^.

We performed an evaluation of these preliminary metrics by inviting a variety of resources to participate in a self-evaluation, where each metric was represented by one or more questions. Nine individuals/organizations responded to the questionnaire, where we emphasized that the objective was not to evaluate their resource, but rather, to evaluate the legitimacy, clarity, and utility of the metrics themselves. This process made it clear that certain metrics (and in some cases, the FAIR Principle underlying it) were not always well-understood. The questionnaire, responses, and evaluation are available in the Zenodo deposit^5^, and a discussion around the responses, what constitutes a "good" answer, and how to quantitatively evaluate an answer, is ongoing, and open to the public on GitHub.

Finally, we envision a framework for the automated evaluation of metrics, leveraging on a core set of existing work and resources that will progressively become part of an open ecosystem of FAIR-enabled (and enabling) tools. Each metric will be self-describing and programmatically executable using the smartAPI^6^ specification, an initiative that extends on the OpenApi specification with semantic metadata. FAIRsharing^7^ will provide source information on metadata, identifier schemas and other standards, which are core elements to many metrics. A “FAIR Accessor”^8^ will be used to publish groups of metrics together with metadata describing, for example, the community to which this set of metrics should be applied, the author of the metrics set, and so on. An application will discover an appropriate suite of metrics, gather the information required by each metric’s smartAPI (through an automated mechanism or through a questionnaire), and then execute the metric evaluation. The output will be an overall score of FAIRness, a detailed explanation of how the score was derived (inputs/outputs for each metric) and some indication of how the score could be improved. Anyone may run the metrics evaluation tool in order to, for example, guide their own FAIR publication strategies; however, we anticipate that community stakeholder organizations and other agencies may also desire to run the evaluation over critical resources within their communities, and openly publish the results. For example, FAIRsharing will also be one of the repositories that will store, and make publicly available, FAIRness grade assessments for digital resources evaluated by our framework, using the core set of metrics.

Measurements of FAIRness are, in our opinion, tangential to other kinds of metrics, such as measurements of openness^9^ or measurements of reuse or citation. While we appreciate the added value that open data provides, we have made it clear that openness is not a requirement of FAIRness^10^, since there are data that cannot be made public due to privacy or confidentiality reasons. Nevertheless, these data can reach a high level of FAIRness by, for example, providing public metadata describing the nature of the data source, and by providing a clear path by which data access can be requested. With respect to reuse and citation, we believe that increasing the FAIRness of digital resources maximizes their reuse, and that the availability of an assessment provides feedback to content creators about the degree to which they enable others to find, access, interoperate-between and reuse their resources. We note, however, that the FAIR-compliance of a resource is distinct from its impact. Digital resources are not all of equal quality or utility, and the size and scope of their audience will vary. Nevertheless, all resources should be maximally discoverable and reusable as per the FAIR principles. While this will aid in comparisons between them, and assessment of their quality or utility, we emphasize that metrics that assess the *popularity* of a digital resource are not measuring its FAIRness. With this in-mind, and with a template mechanism in-place to aid in the design of new metrics, we now open the process of metrics creation for community participation. All interested stakeholders are invited to comment and/or contribute via the FAIR Metrics GitHub site.

## Additional information

**How to cite this article**: Wilkinson, M. D. *et al*. A design framework and exemplar metrics for FAIRness. *Sci. Data* 5:180118 doi: 10.1038/sdata.2018.118 (2018).

**Publisher’s note**: Springer Nature remains neutral with regard to jurisdictional claims in published maps and institutional affiliations.

## Figures and Tables

**Table 1:** The template for creating FAIR Metrics.

FIELD	DESCRIPTION
Metric Identifier	FAIR Metrics should, themselves, be FAIR objects, and thus should have globally unique identifiers.
Metric Name	A human-readable name for the metric
To which principle does it apply?	Metrics should address only one sub-principle, since each FAIR principle is particular to one feature of a digital resource; metrics that address multiple principles are likely to be measuring multiple features, and those should be separated whenever possible.
What is being measured?	A precise description of the aspect of that digital resource that is going to be evaluated
Why should we measure it?	Describe why it is relevant to measure this aspect
What must be provided?	What information is required to make this measurement?
How do we measure it?	In what way will that information be evaluated?
What is a valid result?	What outcome represents "success" versus "failure"
For which digital resource(s) is this relevant?	If possible, a metric should apply to all digital resources; however, some metrics may be applicable only to a subset. In this case, it is necessary to specify the range of resources to which the metric is reasonably applicable.
Examples of their application across types of digital resource	Whenever possible, provide an existing example of success, and an example of failure.
Examples of the application of this table to metric creation are available at https://github.com/FAIRMetrics.	
